# Systemic Lupus Erythematosus (SLE) with Acute Nephritis, Antineutrophil Cytoplasmic Antibody- (ANCA-) Associated Vasculitis, and Thrombotic Thrombocytopenic Purpura (TTP): A Rare Case Report with Literature Review

**DOI:** 10.1155/2019/8750306

**Published:** 2019-12-14

**Authors:** Sohail Farshad, Christopher Kanaan, Solomiia Savedchuk, Dillon S. Karmo, Alexandra Halalau, Abhishek Swami

**Affiliations:** ^1^Beaumont Health, Royal Oak, Department of Internal Medicine, 3601 W 13 Mile Rd, Royal Oak, MI 48073, USA; ^2^Oakland University William Beaumont School of Medicine, 586 Pioneer Dr, Rochester, MI 48309, USA; ^3^Beaumont Health, Royal Oak, Division of Nephrology, 3601 W 13 Mile Rd, Royal Oak, MI 48073, USA

## Abstract

Thrombotic thrombocytopenic purpura (TTP) is a potentially fatal disorder that requires urgent identification and treatment. The association of TTP with systemic lupus erythematosus (SLE) and vasculitis has been reported, however, never simultaneously. A 33-year-old woman with a history of SLE presented with acute abdominal pain, fever, arthralgias, and skin rash. She had acute severe hypertension, diffuse abdominal tenderness, and petechial rash. Diagnostic work-up revealed active urine sediment with proteinuria and hematuria and elevated creatinine, anemia, and thrombocytopenia. She was diagnosed with acute lupus nephritis and early microangiopathic hemolytic anemia in the setting of hypertensive urgency and started on intravenous methylprednisolone 500 mg once a day. Within 48 hours, she developed shock with multiorgan dysfunction and succumbed to her illness. Laboratory tests later showed ADAMTS13 activity less than 10% consistent with TTP and p-antineutrophil cytoplasmic antibody (ANCA) positivity. Autopsy revealed small-vessel vasculitis of the visceral organs. Kidney biopsy demonstrated diffuse proliferative glomerulonephritis. This case illustrates the occurrence of SLE nephritis, p-ANCA vasculitis, and severe TTP with rapidly fatal course, and the importance of having a low threshold for initiating plasma exchange therapy. Here, we discuss the case and provide a literature review on cases of TTP with SLE and vasculitis.

## 1. Introduction

Thrombotic thrombocytopenic purpura is a primary thrombotic microangiopathy that causes microangiopathic hemolytic anemia (MAHA). Congenital and acquired TTP are due to a deficiency of von Willebrand factor (VWF) cleaving protein, also known as ADAMTS13 (a disintegrin and metalloproteinase with a thrombospondin type 1 motif, member 13—von Willebrand factor cleaving protein) [1]. Congenital TTP is due to an inherited deficiency of ADAMTS13, while acquired immune TTP is due to the reduction of ADAMTS13 by autoantibodies directed against ADAMTS13 [1, 2]. In the absence of ADAMTS13, ultralarge multimers of VWF (ULVWF) released from endothelium are not cleaved appropriately and cause spontaneous platelet aggregates in conditions of high shear, such as in the microvasculature of the brain, heart, and kidneys. Although severe deficiency of ADAMTS13 defines TTP (typically, activity <10%), the diagnosis of TTP ultimately relies on clinical judgment since ADAMTS13 measures are often not available for several days, and different assay methods may yield different results [3]. Therefore, the diagnosis of TTP can be difficult at times, as there can be clinical overlap with a spectrum of other pathological processes such as disseminated intravascular coagulation (DIC), infections, and autoimmune disorders.

TTP has been reported to be associated with a variety of autoimmune diseases [4–6]. High level of suspicion and rapid initiation of plasmapheresis, also known as plasma exchange (PEX) are critical as the mortality in TTP before the era of plasmapheresis reached 90% [7]. Even with plasma exchange, SLE-associated TTP has higher mortality of 34–62.5% compared with idiopathic TTP, which is reported to have about 20% mortality [8].

This case report describes a simultaneous manifestation of TTP, SLE flare with nephritis, and ANCA-associated vasculitis (AAV). Based on our literature search, this is the first case to report on these three afflictions occurring at the same time. In addition, we illustrate a literature review on cases of TTP with SLE and TTP with vasculitis.

## 2. Case Details

A 32-year-old African American female with a past medical history of SLE presented to the hospital with epigastric pain, vomiting, subjective fever, arthralgias, and a petechial rash on her palms and soles that started two days after traveling out of United States to the Carribean Islands on a cruise. She was previously treated with hydroxychloroquine and prednisone for SLE but was not on any medications prior to presentation due to loss of follow-up.

Physical exam revealed acute severe hypertension with blood pressure of 187/116 mmHg, normal heart rate, and normal temperature. She was drowsy with altered sensorium but answered questions appropriately; cranial nerve examination was unremarkable. She had a palpable purpuric rash over the palmar and plantar surfaces, as well as the sternum. There was diffuse abdominal tenderness. Laboratory test results upon presentation are shown in Table 1.

Ultrasound of the abdomen revealed an edematous right kidney with mild perinephric fluid without collecting system dilation.

She was diagnosed as having acute lupus nephritis given the findings of active urine sediment, positive dsDNA in high titers, and low complements. Thrombocytopenia was felt to be secondary to SLE flare and/or TMA secondary to severe hypertension. The care team did acknowledge the possibility of TTP due to the presence of thrombocytopenia and MAHA; however, initial suspicion was low as the findings were explained by SLE flare and acute severe hypertension.

After less than 24 hours of hospitalization, the patient developed fever of 102.4 degrees Fahrenheit and had one episode of hematemesis. She was transfused 1 unit of platelets in anticipation for kidney biopsy. She was started on methylprednisolone 500 mg intravenous once a day for acute lupus nephritis. Follow-up troponin was noted to be high at 12 ng/mL. Electrocardiogram was negative for acute ischemia. Her blood pressure increased to 196/139 mmHg, and she was transferred to the medical intensive care unit for further management. Echocardiogram showed no wall motion abnormalities. The next day, she became increasingly lethargic with labored breathing; arterial blood gas showed severe metabolic acidosis, and she became profoundly hypotensive. The patient acutely decompensated requiring vasopressors and intubation. She then developed pulseless electrical activity cardiac arrest and was unable to be resuscitated. An autopsy was requested. Pending serum laboratory tests that resulted after death are shown in Table 2.

Blood cultures were unremarkable for an infectious etiology, including *E. coli*. Autopsy revealed extensive petechial hemorrhages and soft tissue edema surrounding soft tissue of multiple organ systems including small bowel, kidneys, adrenal glands, and spleen. Thrombotic microangiopathy was the underlying pathologic finding seen throughout the internal organs. Vasculitis was seen in the small vessels of the heart, small and medium vessels of the intestines, and the myometrium. Diffuse proliferative glomerulonephritis was also reported.

## 3. Literature Review

Our literature review revealed no published cases of simultaneous TTP, SLE, and vasculitis. Therefore, we separately discuss published cases on TTP with SLE and TTP with vasculitis.

### 3.1. TTP and SLE

A literature search using the databases Pubmed, Web of Science, Cochrane, and Google Scholar identified a review of 105 cases of TTP associated with SLE between 1999 and 2011 [9] and four more recent case reports. Here, we illustrate the findings of one large literature review [9] and provide a table with summary of four more recent case reports [4, 5, 10, 11].

According to the literature review summary by Jiang et al. [9], 82.9% of patients with TTP and SLE were females. The mean age was 32.3 (similar to our patient's age) years with a median of 30 and range of 8 to 74 years. SLE preceded TTP in 50.5% of the cases (as in our case). The most common complaints were fever (19.1%), fatigue (16.2%), and headache (14.7%). In comparison to our patient's presenting symptoms, abdominal pain, rash/ecchymosis/petechiae, and vomiting were reported in only 8.8%, 5.9%, and 1.5% of the patients, respectively. Thrombocytopenia and anemia were reported in 100% and 81.9% of the cases, respectively. Mean platelet counts were 39.3 bil/L. A peripheral blood smear showed schistocytes in all tested cases. Severe deficiency in ADAMTS13 activity (less than 10% of normal) was found in 40.6% of the cases reported. Renal impairment including proteinuria and hematuria and increase in levels of plasma urea nitrogen and creatinine was reported in 83.8% of the cases. Treatment modalities and mortality outcome are reported in Table 3.

In the four individualized case reports [4, 5, 10, 11], treatment plans consisted of either plasma exchange, IV methylprednisolone, oral prednisone therapy, cyclophosphamide, and/or hydroxychloroquine. All patients had resolution of acute TTP crisis as well as a return to baseline of laboratory levels measured. There were no mortalities. We demonstrate each case's treatments and outcomes separately and report them in Table 4.

### 3.2. TTP and Vasculitis

A literature search using the databases Pubmed, Web of Science, Cochrane, and Google Scholar identified 4 case reports [6, 12–14] on patients presenting with TTP and vasculitis, an uncommon association.

All four cases (100%) were female patients with a mean age of 54.75 years (range 17–77). Our patient was considerably younger than the mean age. 75% of patients had some form of sensory disturbance or neurologic deficit, 50% of the patients had fever, and 50% had purpura. Other presenting features included nonbloody diarrhea, menorrhagia, syncope, nausea, vomiting, leg edema, purpura, digestive tract hemorrhage, paralysis, lack of appetite, respiratory distress, headache, and emotional disturbance. Every patient had renal dysfunction. 75% of the patients had significant proteinuria and microscopic hematuria on urinalysis. 50% of cases reported an elevated CRP level. All cases reported an ANCA positive associated vasculitis. 50% of the cases reported a positive myeloperoxidase (MPO) level. Our patient did not have MPO and proteinase 3 antibody level checked.

All patients were treated with plasma exchange and glucocorticoids. Two patients were also treated with cyclophosphamide, and one patient was also treated with rituximab. All patients achieved remission, and there were no mortalities reported thereafter. Treatments and outcomes are reported in Table 5.

## 4. Discussion

Here, we present a unique case that was simultaneously afflicted with rapidly progressive thrombotic thrombocytopenic purpura, lupus flare/nephritis, and ANCA-associated vasculitis in the heart, myometrium, and the bowels, which is the first to be reported. It is possible that this phenomenon is underrecognized, as there have been reported associations between TTP with SLE and TTP with vasculitis, as shown in the literature review.

Given the severity of each disease process individually, it is likely that the simultaneous occurrence of TTP, SLE, and vasculitis is associated with high morbidity and mortality. Our patient's rapid clinical deterioration suggests that this phenomenon is aggressive and can progress rapidly to death. This is supported by the fact that despite PEX, SLE-associated TTP has higher mortality, 34–62.5% compared to idiopathic TTP, which is reported to have about 20% mortality [8]. Often times, the diagnosis is delayed as SLE can mimic clinical and diagnostic manifestations of TTP, which may delay treatment. ADAMTS13 level result often takes several days, contributing to delayed diagnosis and treatment. Also, clinicians delay initiating empiric PEX given that it requires central venous catheter placement, ICU admission, and risk of adverse effects. However, our patient's clinical course suggests that physicians should have a lower threshold to initiate PEX. It is unclear if higher doses of systemic glucocorticoids or initiating other immunosuppressants such as cyclophosphamide have a role in this scenario, and this is an area for further research.

SLE and ANCA-associated vasculitis are recognized as two distinguishable diseases, yet in rare cases such as this one, they do occur simultaneously which is termed SLE/AAV overlap syndrome. Furthermore, those who are found to have both overlapping syndromes commonly present with more severe clinical presentation and especially severe renal disease [15]. Due to the rarity of this SLE/AAV overlap syndrome, its epidemiologic pattern has not yet been well established, but one systematic literature review study reported that patients were mostly female with a wide distribution of ages [16].

The literature review revealed that treatment of TTP and SLE with immunosuppressants only (without PEX) was associated with the highest mortality. Mortality was less with PEX therapy alone and least with PEX and immunosuppressants [9]. In cases of TTP with vasculitis, all cases were treated with at least PEX and glucocorticoids, with all cases achieving clinical remission [6, 12–14]. Clinical judgement and supporting lab results must be used to rule out an infectious process prior to initiating immunosuppressive therapy. Our patient was treated with high-dose corticosteroids within 24 hours, yet her clinical status still rapidly declined.

This case illustrates the importance of strongly considering TTP in a patient who presents with a SLE flare and features consistent with TMA. This is critical, given the paramount importance of timely recognition and initiation of TTP management. A useful and previously validated prediction tool that clinicians should consider using to score-stratify patients with possible thrombotic microangiopathies if timely ADAMTS13 results is not available is the PLASMIC score [17]. The PLASMIC scoring system has demonstrated to be more reliable in its diagnostic accuracy when compared to standard clinical assessment alone [17]. TTP has the highest mortality rate of all thrombotic microangiopathy syndromes, as high as 90 percent when not treated with PEX [7], and a delay in initiating therapy could be fatal.

## 5. Conclusion

This case demonstrates the rapidly progressive and fatal disease syndrome of TTP, SLE, and ANCA-associated vasculitis, all of which have overlapping features. The nonspecific signs and symptoms of TTP may hamper a physician's ability to suspect it on clinical grounds alone, especially in patients with underlying autoimmune conditions. Therefore, when patients with SLE present with thrombocytopenia and features of MAHA, the possibility of TTP should be considered with prompt initiation of empiric plasmapheresis while awaiting test results for confirmation.

## Figures and Tables

**Table 1:** Laboratory test results within 24 hours.

Variable	Result	Reference range, adults
White blood cell (WBC)	5.5 bil/L	3.3–10.7 bil/L
Hemoglobin	12.1 g/dL	12.1–15.0 g/dL
Platelets on presentation	25 bil/L	150–400 bil/L
Platelets 9 hours after presentation	9 bil/L	150–400 bil/L
Creatinine	0.83 mg/dL	0.60–1.40 mg/dL
Total bilirubin	1.8 mg/dL	0.3–1.2 mg/dL
Direct bilirubin	0.6 mg/dL	0.0–0.3 mg/dL
Beta-human chorionic gonadotropin	<2 mIU/mL	0–5 mIU/mL
Erythrocyte sedimentation rate (ESR)	80 mm/hr	0–18 mm/hr
C-reactive protein (CRP)	5.6 mg/dL	0.0–0.8 mg/dL
Lactate dehydrogenase (LDH)	1,139 U/L	100–238 U/L
Haptoglobin	<8 mg/dL	40–240 mg/dL
D-dimer	>9,999 ng/mL	0–499 ng/mL
Prothrombin time (PT)	10.1 seconds	9.3–12.4 seconds
Partial thromboplastin time (PTT)	25.4 seconds	23.0–30.0 seconds
Schistocytes	3–5/hpf	<1/hpf
Fibrinogen	331 mg/dL	175–375 mg/dL
Reticulocytes	85 bil/L	21–100 bil/L
ANA titer	≥1 : 1280	<1 : 160
Anti-double stranded DNA (dsDNA)	1,021 IU/mL	0.0–99.9 IU/mL
Complement C3	54 mg/dL	80–200 mg/dL
Complement C4	7 mg/dL	12–43 mg/dL
Urinalysis protein	>300 mg/dL	0 mg/dL
Urinalysis blood	3+	0
Urinalysis red blood cells (RBC)	>50/hpf, no RBC casts	0–2/hpf

**Table 2:** Laboratory test results after 24 hours.

Variable	Result	Reference range, adults
Antineutrophilic cytoplasmic antibody (ANCA)	p-ANCA 1 : 640	<1 : 20
ADAMTS13	<10%	>50%
Antistreptolysin O	399 IU/mL	0–199 IU/mL
Antiphospholipid panel	Anticardiolipin IgA, IgG negative Beta-2 glycoprotein 1 IgM, IgG negative Anticardiolipin IgM 13.5 MPL : intermediate	<12.5 MPL negative 12.5–20 MPL intermediate >20 MPL low-medium to high positive
Paroxysmal nocturnal hemoglobinuria flow cytometry	Negative	Negative
HIV, EBV IgM, dengue IgG and IgM, and hepatitis A, B, and C virus	Negative	Negative
Blood cultures	Negative	Negative

Ig, immunoglobulin; HIV, human immunodeficiency virus; EBV, Epstein–Barr Virus.

**Table 3:** Treatment modalities and mortality.

Treatment modalities	Mortality
Plasma exchange (PEX) alone	16.7% (1/6)
Glucocorticoids (GC) and/or cytotoxics (including cyclophosphamide, mycophenolate mofetil, vincristine, cyclosporine A, and methotrexate) without PEX	30% (3/10)
PEX + GC	11.4% (4/35)
PEX + cytotoxics	0% (0/3)
PEX + GC + cytotoxics	9.6% (5/52)
Rituximab + PEX with or without GC or cytotoxics	0% (0/11)

**Table 4:** TTP and SLE treatments and outcomes.

Author	Age	Treatment	Outcome
Changcharoen and Bolger [4]	35 years	Plasma exchange Glucocorticoids Hydroxychloroquine Mycophenolate	Resolution of acute symptoms Clinical and laboratory findings back to baseline Clinical remission achieved
Bamidele et al. [5]	58 years	Glucocorticoids Hydroxychloroquine	Resolution of TTP specific acute symptoms Lupus exacerbation Clinical remission status unknown
Abu-Hismeh et al. [10]	34 years	Plasma exchange Glucocorticoids Rituximab Cyclophosphamide	Unresponsive to plasma exchange, glucocorticoids, and rituximab After cyclophosphamide, achieved clinical and laboratory findings back to baseline Clinical remission achieved
Blum and Blake [11]	44 years	Plasma exchange Glucocorticoids Mycophenolate Cyclophosphamide	Hemolytic parameters nearly or completely normalized Active inflammation markers decreased Remained dependent on dialysis Clinical remission achieved

**Table 5:** TTP and vasculitis treatments and outcomes.

Author	Age	Treatment	Outcome
Argawal et al. [6]	17 years	Plasma exchange Glucocorticoids Cyclophosphamide	Return to baseline kidney function Clinical remission achieved
Nagai et al. [12]	77 years	Plasma exchange Glucocorticoids	Clinical and laboratory findings back to baseline Clinical remission achieved
Yamauchi et al. [13]	59 years	Plasma exchange Glucocorticoids	Clinical and laboratory findings back to baseline Clinical remission achieved
Asamiya et al. [14]	66 years	Plasma exchange Glucocorticoids Cyclophosphamide Rituximab	Hemolytic parameters nearly or completely normalized Remained dependent on dialysis Clinical remission achieved
